# Implementation of a Urinary Tract Infection Treatment Protocol to Improve Prescribing Practices in the Long-Term Care Facility of a Veteran’s Healthcare System

**DOI:** 10.3390/pharmacy8030129

**Published:** 2020-07-24

**Authors:** Spencer H. Durham, Natalie S. Hohmann, Addison H. Ragan

**Affiliations:** 1 Department of Pharmacy Practice, Harrison School of Pharmacy, Auburn University, Auburn, AL 36849-5341, USA; nsh0010@auburn.edu; 2 Department of Pharmacy, Central Alabama Veterans Health Care System, Montgomery, AL 36109, USA; addison.holder@va.gov

**Keywords:** urinary tract infection, long-term care facility, antimicrobial stewardship, appropriate prescribing, antimicrobial resistance, geriatrics, asymptomatic bacteriuria, older adult

## Abstract

Urinary tract infections (UTIs) are a commonly diagnosed problem in long-term care facilities (LTCFs), but antimicrobial treatment is often incorrectly prescribed. Although bacterial resistance to antimicrobials commonly used for UTIs, such as trimethoprim/sulfamethoxazole and fluoroquinolones, has been dramatically increasing, they are still commonly prescribed. The purpose of this project was to determine if implementation of a standard treatment protocol for UTIs, which emphasized correct UTI diagnosis and use of nitrofurantoin and cefpodoxime/ceftriaxone as empiric therapy per the institutional antibiogram, changed clinician prescribing practices. This quasi-experimental model utilized two years of pre-intervention and two years of post-intervention data. Three hundred patient encounters were included. Antibiotics prescribed in the pre-intervention period included: trimethoprim/sulfamethoxazole (32%), ciprofloxacin (14%), amoxicillin (13%), levofloxacin (9%), cefpodoxime (9%), ceftriaxone (8%), amoxicillin/clavulanate (5%), nitrofurantoin (4%), and other (6%). By contrast, antibiotics prescribed in the post-intervention period included: cefpodoxime (46%), nitrofurantoin (30%), ceftriaxone (10%), trimethoprim/sulfamethoxazole (8%), amoxicillin/clavulanate (1%), and other (5%). These differences in prescribed drug between the pre-intervention and post-intervention encounters were statistically significant (*p* < 0.001). Overall, appropriate empiric treatment was prescribed in only 48/217 encounters (22%) during the pre-intervention period, but this increased to 73/83 encounters (88%) in the post-intervention period (*p* < 0.001). The results indicate that the treatment protocol was successful in changing prescribing practices and decreasing the use of inappropriate antimicrobials at the LTCF.

## 1. Introduction

Infections are a common diagnosis in long-term care facilities (LTCFs), with as many as three million infections diagnosed each year [1]. As such, antibiotics are some of the most frequently prescribed drugs in this setting. The Centers for Disease Control and Prevention (CDC) estimate that up to 70% of LTCF residents receive at least one course of an antibiotic in a one-year period [2]. More troubling is the fact that 40–75% of these antibiotics are either unnecessary or inappropriate. Misuse of antimicrobial agents in this patient population can have many untoward consequences, including increased antimicrobial resistance, adverse effects, a greater likelihood of drug–drug interactions, and *Clostridioides difficile* infection [3].

Urinary tract infections (UTIs) are one of the most common infections diagnosed in LTCFs, accounting for up to 20% of infections [4]. Patients in LTCFs may be at a higher risk of UTIs for a variety of reasons, including advanced age, underlying immune suppression, and other disease states that may predispose to UTIs, such as benign prostatic hyperplasia [4,5]. Another major risk factor for UTIs in this population is the presence of an indwelling urinary catheter. Up to 10% of LTCF patients have a urinary catheter in place, often for years at a time. Almost all patients with indwelling urinary catheters become bacteriuric within one month of catheter insertion, although bacteriuria is often not associated with infection [6]. 

Several factors complicate the treatment of UTIs in the LTCF population [4,7]. First, many patients may have asymptomatic bacteriuria (ASB) and do not require treatment with antimicrobials. Additionally, placement of an indwelling urinary catheter may contribute to ASB. In LTCF patients experiencing true infection, appropriate treatment is not well-studied, no consensus treatment guidelines are available, and the appropriate length of treatment is not known. Male patients who develop UTIs are a particularly under-studied group, as are older adults. Therefore, treatment is often based on clinical experience and theoretical extrapolation from well-studied populations, such as otherwise healthy, pre-menopausal females. Additionally, antimicrobial resistance among uropathogens, such as *Escherichia coli*, *Klebsiella pneumoniae*, and *Proteus mirabilis*, is increasing rapidly to drugs historically used for the treatment of UTIs, such as trimethoprim/sulfamethoxazole and the fluoroquinolones, ciprofloxacin and levofloxacin [8,9,10,11,12]. 

Over the last several years, our institution has mimicked the global increase in antimicrobial resistance to these common uropathogens, particularly *E. coli*. On the 2017 LTCF antibiogram, *E. coli* demonstrated only 45% and 18% susceptibility to trimethoprim/sulfamethoxazole and ciprofloxacin/levofloxacin, respectively. Despite this high resistance, clinical pharmacists working in the LTCF noted that trimethoprim/sulfamethoxazole and fluoroquinolones seemed to be the most commonly prescribed empiric therapy for UTIs. In contrast, *E. coli* displayed high sensitivity to nitrofurantoin and ceftriaxone at 96% and 92%, respectively. However, these agents were only occasionally being used. Additionally, there appeared to be no discernable pattern to when prescribers would select one agent for use over another. Finally, in patients with indwelling catheters, it appeared that, in many instances, the catheters were not being replaced in response to suspicion of an infection. 

Because of these discrepancies in the treatment of UTIs, pharmacists sought to improve UTI treatment as an antimicrobial stewardship initiative through the creation of a UTI treatment protocol. The protocol was designed to assist providers in determining when antimicrobial therapy was indicated and to help select appropriate empiric antimicrobials. The primary objective of this quality improvement project was to determine if clinicians accurately followed the protocol, with the goal of decreasing the use of inappropriate empiric antimicrobial therapy for the treatment of UTIs. A secondary outcome was to evaluate if the protocol resulted in indwelling catheters being changed appropriately in patients suspected of experiencing a UTI. 

## 2. Materials and Methods 

This quality improvement project was designated “non-research” by the Central Alabama Veterans Health Care System and was approved by the institutional review board of Auburn University, and this project received no external funding. The LTCF is part of a large Veterans Affairs (VA) Healthcare System and has approximately 107 beds.

### 2.1. Protocol Development

The protocol was developed by the pharmacy department based upon a review of three years of antimicrobial resistance patterns on the LTCF antibiogram combined with current prescribing practices. Antimicrobial stewardship principles focusing on appropriate antibiotic use guided the development of the protocol, and input was obtained from prescribers within the LTCF, including an infectious disease physician, geriatrician, and nurse practitioners. The treatment protocol developed and implemented can be seen in Appendix A. The protocol is divided into two sections: patients with an indwelling catheter and those without. Emphasis is placed on correctly identifying a UTI through the assessment of clinical signs versus laboratory results alone in an effort to decrease the treatment of ASB. However, given that many patients in the LTCF setting may not exhibit traditional signs and symptoms of a UTI, the protocol also recommends obtaining urinalyses and urine cultures for all patients as an objective measure of determining if a UTI is present. In patients with an indwelling catheter who have clinical suspicion of a UTI, the protocol recommends replacing the indwelling catheter and obtaining the urinalysis and urine culture from the new catheter as a means to help distinguish patients with a true infection from those exhibiting bacteriuria from the catheter.

In determining the most appropriate drug therapy to recommend in patients requiring empiric treatment, trends in the institutional antibiogram over time were examined. While it was noted that resistance of uropathogens to trimethoprim/sulfamethoxazole and the fluoroquinolones was dramatically increasing over time, nitrofurantoin and third-generation cephalosporins retained high susceptibility (>90%). Historically, nitrofurantoin was not used often in the LTCF setting due to a long-standing recommendation not to administer the drug to patients with a creatinine clearance (CrCL) less than 60 mL/min [13]. Original studies with nitrofurantoin suggested that it was not excreted in the urine in patients with low CrCL, and could therefore not adequately treat a UTI, while predisposing to adverse effects of the drug. However, recent evidence demonstrated that nitrofurantoin is both safe and effective in patients with a CrCL of ≥ 30 mL/min [14,15]. In 2015, the Beers Criteria amended their recommendations on nitrofurantoin use in older adults to reflect this most recent evidence [13,16]. From an antimicrobial stewardship standpoint, nitrofurantoin is an attractive option for the treatment of UTIs due to its broad-spectrum of activity, distribution predominantly into the urine, and low risk of serious adverse effects [13]. Therefore, the protocol recommends nitrofurantoin as the first-line agent for empiric use for patients with an eGFR of > 40 mL/min. The eGFR was chosen for inclusion on the protocol as opposed to the CrCL because the electronic health record utilized in VA facilities includes the eGFR in laboratory assessments, whereas clinicians would have to calculate the CrCL. For patients with an eGFR < 40 mL/min or those with systemic signs and symptoms of infection, a third-generation cephalosporin (cefpodoxime or ceftriaxone) is recommended as a first-line therapy. The protocol does not include trimethoprim/sulfamethoxazole or the fluoroquinolones as empiric treatment options. 

### 2.2. Study Design

The protocol was approved by the Pharmacy & Therapeutics (P&T) committee of the healthcare system and was officially implemented on 1 September 2017. Prior to the implementation, pharmacy staff provided inservices to prescribers in the LTCF, which include primarily one geriatrician and two nurse practitioners, to help familiarize them with the new protocol and the reason the changes were implemented. A quasi-experimental design was developed to analyze the results of the protocol, with the pre-assessment period of 1 September 2015 to 31 August 2017, and a post-assessment period of 1 September 2017 to 31 August 2019. All data were collected through a retrospective chart review after the end of the post-assessment period. All patients who received any antibiotic for treatment of a UTI during the specified time periods were included in the analysis, and no patients were excluded. Among these patients, each separate UTI infection treated with at least one antibiotic was included and regarded as a UTI “encounter”. 

### 2.3. Data Analysis Plan

Data were analyzed on the encounter level. Descriptive statistics included frequencies, percentages, means, and standard deviations. Patient characteristics (age, sex, and race/ethnicity), encounter characteristics (such as presence of a catheter and presence of local or systemic symptoms), and the percentage of encounters prescribed appropriate empiric therapy were compared between the pre- and post-intervention periods. These bivariate comparisons used independent sample t-tests for continuous variables and Chi-square or Fisher’s Exact for categorical variables. To investigate how the intervention, patient characteristics, and encounter characteristics may predict appropriate vs. inappropriate prescribing of empiric UTI treatment while controlling for patient identity, logistic regression models were performed. Average marginal effects were calculated for predictors in the adjusted models. All analyses were performed with SPSS version 26 (IBM, Armonk, New York, NY, USA) and STATA version 16 (StataCorp LLC, College Station, Texas, TX, USA). 

## 3. Results

### 3.1. Characteristics of UTI Patient Encounters

In total, 300 patient encounters were included over the four years of the study, including 217 encounters in the pre-intervention period and 83 in the post-intervention period. Among the 217 patient encounters analyzed in the pre-intervention and 83 patient encounters analyzed in the post-intervention, most encounters were in male patients (n = 209 [96%] vs. n = 80 [96%], respectively), patients of African American (n = 159 [73%] vs. 70 [84%]) or White race (n = 58 [27%] vs. n = 13 [16%]), and with a mean age of 71.1 years (SD = 10.49) vs. 73.5 years (SD = 10.58) (age was recorded at the patient’s first encounter during the four-year study period). These demographic differences in race/ethnicity between the pre- and post-intervention period encounters were statistically significant (*p* < 0.05); age and sex were not statistically significantly different (both *p* > 0.05). 

### 3.2. Appropriateness of Empiric Treatment

**Figure A1:**
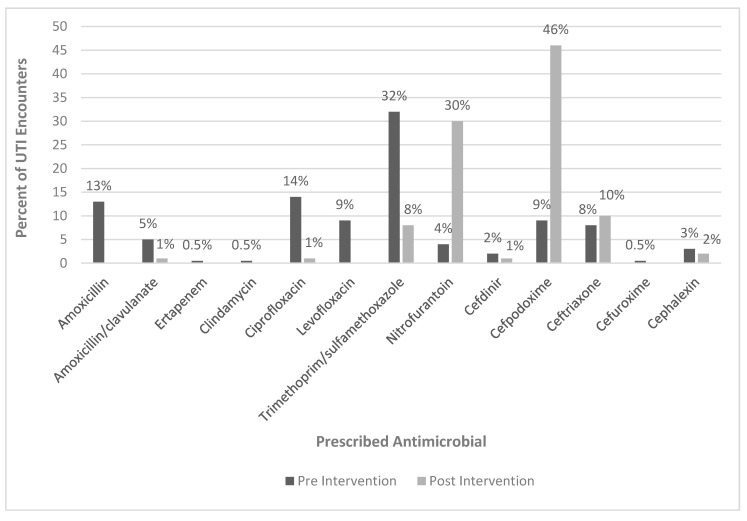
Comparison of prescribed antimicrobial in the pre- and post-intervention period.

Ciprofloxacin was prescribed for 30 pre-intervention encounters (14%) and one post-intervention encounter (1%), while levofloxacin was prescribed in 20 pre-intervention encounters (9%) and 0 post-intervention encounters (Table 1). Trimethoprim/sulfamethoxazole was prescribed in 69 pre-intervention encounters (32%) and 7 post-intervention encounters (8%). Most other encounters received nitrofurantoin (pre = 4%; post = 30%), cefpodoxime (pre = 9%; post = 46%), ceftriaxone (pre = 8%; post = 10%), amoxicillin (pre = 13%; post = 0), or amoxicillin/clavulanate (pre = 5%; post = 1%). These differences in prescribed drug were statistically significant (*p* < 0.001). A comparison of the antibiotics prescribed in both the pre- and post-intervention periods can be seen in Appendix B (Figure A1). 

In addition, 134 encounters (62%) that were treated for UTI had catheters in the pre-intervention period vs. 34 (41%) in the post-intervention period (Table 1); among those encounters with a catheter present, 17 encounters (13%) had the catheter changed due to suspected UTI in the pre-intervention period vs. 10 (29%) in the post-intervention period. In addition, 209 encounters (96%) had urinalyses obtained in the pre-intervention period vs. 73 (90%) in the post-intervention period, while 198 encounters (91%) had urine cultures obtained in the pre-intervention period vs. 71 (87%) in the post-intervention period. These differences between the pre and post encounters were statistically significant for the presence of catheters, changing the catheter when UTI was suspected, and conducting a urinalysis (*p* < 0.05); there was no statistically significant difference in conducting a culture (*p* > 0.05). Further, the duration of antimicrobial therapy ordered was statistically significantly different between the pre-intervention and post-intervention periods (*p* < 0.001), ranging 5-14 days in the pre-intervention period (most often 10 days [57%]) and 7–10 days in the post-intervention period (most often 7 days [86%]). 

Overall, bivariate results show that the appropriate empiric treatment was prescribed in only 48 encounters (22%) during the pre-intervention period, but this increased to 73 encounters (88%) in the post-intervention period. This difference was statistically significant (*p* < 0.001). 

The results of adjusted logistic regression models with average marginal effects show that, during the post-intervention period, UTI patient encounters were 66.8% more likely to be prescribed appropriate empiric treatment compared to UTI encounters during the pre-intervention period (Table 2). This difference was statistically significant (*p* < 0.001). This indicates the treatment protocol is largely being followed, and it was effective in changing prescribing practices and decreasing the use of inappropriate antimicrobials at the LTCF. For every one-year increase in patient age, the probability of prescribing an appropriate empiric therapy decreased by 0.4%; however, this did not reach statistical significance (*p* > 0.05). Because this sample was predominantly composed of male African American patients, sex and race/ethnicity were not included in the final model and thus could not be assessed as independent predictors of appropriate empiric therapy. The presence of local signs/symptoms, systemic signs/symptoms, or an indwelling catheter and conducting a urinalysis or culture were not statistically significant predictors of appropriate empiric therapy (all *p* > 0.05). 

## 4. Discussion

The correct diagnosis and treatment of UTIs in patients who reside in LTCFs is challenging, and the literature to help improve practices is limited. Clinical practice guideline recommendations are not available for this population, which makes the selection of appropriate antimicrobial therapy challenging. Antimicrobial resistance to drugs historically used for the treatment of UTIs, such as trimethoprim/sulfamethoxazole and the fluoroquinolones, is increasing, and may vary widely by location [11]. At our institution, these agents have traditionally been the most prescribed empiric options for the treatment of UTIs, but resistance is such that they are no longer appropriate for empiric use. Therefore, our treatment protocol focuses on the empiric use of agents with high susceptibility, specifically nitrofurantoin and the third-generation cephalosporins. Our project demonstrates that implementation of this UTI treatment protocol was successful in changing prescribing habits of practitioners as evidenced by the dramatically decreased usage of trimethoprim/sulfamethoxazole and the fluoroquinolones and the increased utilization of nitrofurantoin and third-generation cephalosporins. Bivariate results were consistent with adjusted logistic regression model results, showing that UTI encounters after the intervention were more likely to receive appropriate empiric therapy. Additionally, the recommended durations of therapy were largely followed in the post-intervention period, unlike in the pre-intervention period when a wide variety of therapy durations was used for different drugs. Indwelling catheters were also more likely to be changed in the post-intervention period. In the post-intervention period, there was an overall decrease in the number of UTI treatments compared to the pre-intervention period. While other factors may have contributed, the protocol was likely a major factor in the overall reduction. 

Numerous reports in the literature describe antimicrobial stewardship interventions to decrease inappropriate antimicrobial prescribing for UTIs. Many studies have involved educational interventions in the acute care setting [17,18,19,20]. Data are less robust in the LTCF setting, with only a few studies that can be reasonably compared to ours. Trautner and colleagues described an intervention conducted at two VA health systems to reduce the number of urine cultures obtained in an effort to reduce overall antimicrobial prescribing [21]. The patient population included patients with urinary catheters on the acute care medicine wards and residents of the LTCFs. The intervention included case-based audit and feedback, as well as an algorithm to aid in the diagnosis of a UTI. The overall rate of ordering urine cultures significantly decreased during the intervention period, as did antimicrobial prescribing for UTIs (*p* < 0.001 for both). Zabarsky and colleagues evaluated the use of an education intervention to decrease both urine culture collection and antimicrobial treatment of UTIs in a LTCF [22]. The education interventions included educating nurses not to collect urine cultures in the absence of UTI symptoms and educating primary care providers not to prescribe treatment for ASB. In the six-month period after the intervention began, urine culture collection decreased significantly (*p* < 0.0001) as did treatment of ASB (*p* < 0.0017) compared to the three-month period prior to the intervention. Additionally, the reductions persisted for 30 months after the start of the intervention. As part of the implementation of our protocol, education regarding appropriate treatment of both ASB and UTIs was provided to the primary prescribing clinicians. In most of the studies described in the literature, the treatment algorithm or protocol focused primarily on whether or not to prescribe an antibiotic. Unlike previous literature, our protocol incorporated recommendations to reduce inappropriate prescribing in addition to recommendations of selecting a specific empiric antibiotic for those patients with true suspicion of UTI. 

Although the protocol can largely be considered a success, areas for improvement were noted. In particular, the use of cephalosporins was questionable in a number of cases, and these patients likely could have been placed on nitrofurantoin empirically. Although cephalosporins have high susceptibility based on the antibiogram, the protocol is designed to limit their use as they are associated with untoward adverse effects, such as the development of *C. difficile* infection and increased incidence of extended-spectrum beta-lactamase (ESBL) producing organisms. Therefore, the protocol limits the use of these agents to patients with systemic infections or those with poor renal function. In some cases where cephalosporins were used, there was no clear documentation of systemic signs and symptoms of infection. In other cases, prescribers sometimes considered non-definitive parameters to be signs/symptoms of systemic infection, such as abdominal pain, which may be unrelated to a UTI. In other instances, the only documented abnormality in the patient was “cloudy urine” and a cephalosporin was used for empiric treatment. In addition, although the protocol resulted in a significant increase in appropriately changing urinary catheters, more than two-thirds of patients who should have received a catheter change did not. Finally, the prescribing habits of individual providers was not formally assessed, although there did not appear to be one outlier prescriber but rather collective inappropriate prescribing across the three primary providers within the LTCF. Efforts are currently underway to provide further education to the LTCF practitioners on the appropriate use of cephalosporins and the importance of changing indwelling catheters in patients suspected of having a UTI.

The evaluation of this project does have certain limitations that must be addressed. First, this was a retrospective chart review with limitations inherent to this type of study design. Part of the purpose of implementing the protocol was to decrease the number of patients being inappropriately treated for UTIs, which is why the protocol emphasizes obtaining urinalysis results first, prior to initiation of antimicrobial therapy. Although there were fewer courses of therapy in the post-intervention period, it cannot be conclusively determined if this was due to practitioners obtaining urinalyses but not prescribing antibiotics. Because urinalyses are ordered for reasons besides assessment of infection, patients had to be identified for inclusion based on receipt of antibiotic therapy rather than performing a chart review on all patients for whom a urinalysis was obtained. In addition, hospitalizations and deaths from UTIs were not specifically assessed as part of this project. Finally, the ultimate goal of changing prescribing practices would be to see changes reflected in the institutional antibiogram. However, changes in an antibiogram often take years to reflect, so this could not be adequately assessed during the study period, but it will be assessed in the future. 

## 5. Conclusions

The implementation of this UTI treatment protocol was successful and resulted in more appropriate empiric treatment of UTIs. Use of agents with poor susceptibility to uropathogens at our facility, such as trimethoprim/sulfamethoxazole and the fluoroquinolones, decreased after implementation of the protocol, while use of agents with excellent susceptibility, such as nitrofurantoin and cefpodoxime, increased. The use of a standard treatment protocol for UTIs should be considered in other LTCFs as an antimicrobial stewardship initiative to improve empiric antibiotic use. 

## Figures and Tables

**Table 1:** Encounter characteristics ^1^.

Characteristic	Total Encounters	Pre-Intervention	Post-Intervention	*p*-Value
	n = 300 ^1^	n = 217	n = 83	
	n (%)*	n (%)*	n (%) *	
**Local Signs/Symptoms**				<0.001 **
Yes	32 (11%)	13 (6%)	19 (23%)	
No	268 (89%)	204 (94%)	64 (77%)	
**Systemic Signs/Symptoms**				0.013 **
Yes	59 (20%)	35 (16%)	24 (29%)	
No	241 (80%)	182 (84%)	59 (71%)	
**Catheter Present**	168 (56%)	134 (62%)	34 (41%)	0.001 **
Yes	132 (44%)	83 (38%)	49 (59%)	
No				
**Catheter Changed** **^2,3^**				<0.001 **
Yes	27 (16%)	17 (13%)	10 (29%)	
No	141 (84%)	117 (87%)	24 (71%)	
**Urinalysis Performed** **^2^**				0.035 **
Yes	282 (95%)	209 (96%)	73 (90%)	
No	16 (5%)	8 (4%)	8 (10%)	
**Culture Conducted** **^2^**				0.232
Yes	269 (90%)	198 (91%)	71 (87%)	
No	30 (10%)	19 (9%)	11 (13%)	
**Cultured Species** **^2,4^**				0.344
*Citrobacter freundi/koseri/yougae*	3 (2%)	2 (3%)	1 (2%)	
*Diptheroids*	2 (2%)	0	2 (4%)	
*Enterobacter cloacae*	4 (3%)	4 (5%)	0	
*Enterococcus faecalis/faecium*	6 (5%)	3 (4%)	3 (7%)	
*Escherichia coli*	30 (24%)	17 (22%)	13 (28%)	
*Klebsiella pneumoniae*	12 (10%)	6 (8%)	6 (13%)	
*Proteus mirabilis/hauseri*	23 (19%)	14 (18%)	9 (20%)	
*Providencia stuartii*	1 (1%)	1 (1%)	0	
*Pseudomonas aeruginosa*	4 (3%)	3 (4%)	1 (2%)	
*Staphylococcus aureus/epidermidis/haemolyticus*	6 (5%)	6 (8%)	0	
*Viridans streptococcus*	1 (1%)	0	1 (2%)	
Yeast	1 (1%)	1 (1%)	0	
Two microbes	27 (22%)	18 (39%)	9 (20%)	
**Prescribed Antimicrobial**				<0.001 **
Amoxicillin	29 (10%)	29 (13%)	0	
Amoxicillin/clavulanate	12 (4%)	11 (5%)	1 (1%)	
Ertapenem	1 (0.3%)	1 (0.5%)	0	
Clindamycin	1 (0.3%)	1 (0.5%)	0	
Ciprofloxacin	31 (10%)	30 (14%)	1 (1%)	
Levofloxacin	20 (7%)	20 (9%)	0	
Trimethoprim/sulfamethoxazole	76 (25%)	69 (32%)	7 (8%)	
Nitrofurantoin	33 (11%)	8 (4%)	25 (30%)	
Cefdinir	6 (2%)	5 (2%)	1 (1%)	
Cefpodoxime	57 (19%)	19 (9%)	38 (46%)	
Ceftriaxone	25 (8%)	17 (8%)	8 (10%)	
Cefuroxime	1 (0.3%)	1 (0.5%)	0	
Cephalexin	8 (3%)	6 (3%)	2 (2%)	
**Duration of Treatment Ordered** **^2^**				<0.001 **
5 days	13 (4%)	13 (6%)	0	
7 days	146 (49%)	75 (35%)	71 (86%)	
10 days	136 (45%)	124 (57%)	12 (14%)	
14 days	4 (1%)	4 (2%)	0	
**Empiric Treatment Appropriate**				<0.001 **
Yes	121 (40%)	48 (22%)	73 (88%)	
No	179 (60%)	169 (78%)	10 (12%)	

^1^ There were 300 patient encounters, across the four-year study period. Twenty-one patients who had at least one encounter in the pre-intervention period also had at least one encounter in the post-intervention period. ^2^ There are some missing data: catheter changed n = 168 encounters (134 pre-intervention and 34 post-intervention); urinalysis conducted n = 298 encounters (217 pre-intervention and 81 post-intervention); culture conducted n = 299 encounters (217 pre-intervention and 82 post-intervention); cultured species n = 123 encounters (77 pre-intervention and 46 post-intervention); duration of treatment ordered n = 299 encounters (216 pre-intervention and 83 post-intervention). ^3^ Among those encounters with an indwelling catheter present. ^4^ Among cultures conducted, with growth, and judged uncontaminated. * Percentages may not add to 100 due to rounding. ** *p* < 0.05, statistically significant.

**Table 2:** Adjusted logistic regression model results predicting appropriateness of empiric treatment for UTI in a long-term care facility of a veteran’s healthcare system ^1^.

Predictors	Average Marginal Effect	*p*-Value
**Intervention** Post Pre (ref)	0.668	<0.001 *
**Age** (years)	−0.004	0.098
**Local signs/symptoms** Yes No (ref)	0.147	0.124
**Systemic signs/symptoms** Yes No (ref)	0.064	0.309
**Catheter present** Yes No (ref)	−0.030	0.531
**Urinalysis obtained** Yes No (ref)	0.179	0.078
**Culture conducted** Yes No (ref)	0.021	0.845

^1^ Results of final logistic regression models with average marginal effects, n = 298. The outcome variable was dichotomized based on appropriateness of the empiric treatment according to the UTI protocol (appropriate vs. not appropriate). Intervention status (pre vs. post) was included in the model as a predictor variable. Patient and encounter characteristics were also included as predictor variables, including age at first encounter, presence of local signs/symptoms, presence of systemic signs/symptoms, presence of an indwelling catheter, urinalysis conducted, and culture conducted. As this sample was predominantly made up of male African American patients, sex and race/ethnicity were not included in the final model. In addition, because of the relatively large amount of missing data for the culture species, this was not included in the final model. Patient ID was controlled for as a covariate to account for correlation due to patients having multiple encounters over the study period. * *p* < 0.05, statistically significant.

## Appendix B

Differences in prescribed antimicrobial between the pre- and post-intervention period were statistically significant (*p* < 0.001).
